# Tau *GST*s involved in regulation of leaf abscission by comparison the gene profiling of *MeGST*s in various abscission-promoting treatments in cassava abscission zones

**DOI:** 10.1186/s12863-018-0627-6

**Published:** 2018-07-13

**Authors:** Wenbin Liao, Shuxia Li, Cheng Lu, Ming Peng

**Affiliations:** Institute of Tropical Bioscience and Biotechnology, Chinese Academy of Tropical Agricultural Sciences, ITBB, CATAS, Xueyuan Rd No 4, Haikou City, Hainan Province People’s Republic of China 571101

**Keywords:** Ethylene treatment, Drought treatment, *GST* gene family, Transcriptome analysis, Cassava abscission zone

## Abstract

**Background:**

Glutathione S-transferases (GSTs) have been reported to regulate the plant tolerance to environmental stresses. Many plant GSTs exhibited the roles on promoting tolerance to drought stress, oxidative stress and plant hormones. The biological function of GSTs has been well characterized in *Arabidopsis thaliana* in response to exogenous environmental stresses. However, their regulation function under exogenous environmental stresses regulating leaf abscission in cassava (*Manihot esculenta* Crantz) remained unknown.

**Results:**

Here, 83 GSTs were identified from tropical plant cassava. The amino acid motifs and phylogenetic analyses indicated that MeGSTs were divided into 9 classes. The global expression analyses were carried out to analyze the gene expression patterns of *MeGST* in cassava abscission zones by comparing the *MeGST* genes expression patterns in both ethylene and drought induced cassava leaf abscission. Totally, 34 *GST*s were detected to express in both ethylene and drought induced leaf abscission in cassava abscission zones. Comparison of *GST* expression profiling between ethylene and drought induced leaf abscission suggested that Tau *GST* genes showed with the similar expression in both treatments induced leaf abscission in cassava abscission zone. GO annotation indicated that all 17 Tau *GST* genes participated in the pathway of toxin catabolism (GO: 0009407). The expression levels of 17 Tau *MeGST* genes were analyzed in two cassava cultivars, ‘SC124’ and ‘Arg7’, the two cultivars exhibit different levels of leaf abscission when suffered from the same environmental stress. Higher expression levels of Tau *MeGST*s were detected in the precocious abscission Arg7 cultivar, while lower expression levels in delayed abscission SC124 cultivar. All the results indicated that Tau *MeGSTs* have the function in regulation the cassava leaf abscission under environmental stresses.

**Conclusion:**

Analysis of the expression patterns of *GST*s in various abscission-promoting treatments in cassava abscission zones helps us to understand the possible roles of *GST*s in cassava leaf abscission.

**Electronic supplementary material:**

The online version of this article (10.1186/s12863-018-0627-6) contains supplementary material, which is available to authorized users.

## Background

Glutathione S-transferases (GSTs; EC 2.5.1.18), as detoxification enzymes, are widely presented in plants, bacteria, fungi and animals [1]. GSTs have the ability to detoxicate the cytotoxic compounds produced by environmental stresses [1]. Generally, a conserved GSH binding site (G-site) located in the N-terminal domain of GSTs, and an electrophilic substrate binding site (H-site) in C-terminal domain of GSTs [1]. In *Arabidopsis*, GSTs can be divided into eight subfamilies based on amino acid sequence similarity, including Phi, Tau, Theta, Zeta, Lambda, DHAR, TCHQD and MAPEG [1, 2]. Since then several new classes were added to the GST protein family, such as EF1Bγ and GHR class GSTs [3, 4]. Phi and Tau are the two largest plant-specific GSTs; these GSTs can regulate stress responses [1, 2]. Up to now, numerous *GST* gene family members have been identified in various species by genome-wide analysis, i.e., 81 *GST* genes in *Populus* [5], 55 *GST* genes in *Arabidopsis* [6], 79 in rice [7, 8], 84 in barley [9], 23 in sweet orange [10], 27 in Japanese larch [1, 10], and 59 and 49 in the *G. raimondii* and *G. arboreum* genome [1].

Many plant GSTs exhibited peroxidase activity and played function in promoting tolerance to oxidative stress, osmotic dehydration and plant hormones [1, 2]. In *Arabidopsis*, Phi *GST* 9 (*GSTF9*) was induced by salt and salicylic acid responses that acted the function in regulating redox homeostasis [11]. *GST11* was discovered to regulate the plants tolerance to oxidative stress, metal toxicities and extreme of temperature [12]. Four Phi *GST*s displayed to participate in plant stress regulation by co-silencing of multiple genes to alter metabolic sensitivity of the plants to oxidative stress [6]. Tau *GST8* was up-expressed by oxidative stress, exhibiting the gene has the function to regulate oxidative reaction in stressed plants [13]. Two classes of *GST*s (DHARs and GSTLs) were induced by chemicals and oxidative stresses, exhibiting the function to regulate redox homeostasis in stressed plants [14]. *GSTU19* was induced by salt and drought; exhibiting an increased activity of antioxidant enzymes and the level of proline in transgenic plants [2]. *GSTU17* can be induced by phytohormones [15]. The GSTF2 protein can be up-regulated by ethylene treatment [16]. The *GST*s can be induced by allyl isothiocyanate at lower doses; the up-regulation of GSTs was proved to promote the tolerance to oxidative stress [17]. In rice, over expression of *GSTU4* has been proved to improve resistance to oxidative stress and salinity stress [18]. In soybean, *GSTL1* was induced by salt treatment; over expression this gene markedly decreased the accumulation of reactive oxygen species in transgenic plants under salt stress [19]. In *Pyrus pyrifolia*, one *GST* was induced by abiotic stress, transgenic lines showed the ability to enhance tolerance to oxidative damage [20]. In *Salicornia brachiata*, *GSTU* can be induced by various abiotic stressors [21].

Cassava (*Manihot esculenta* Crantz) plants have the ability to adapt to new stress conditions by shedding leaves at their petioles when the plants suffer from the adverse environmental stresses [22], which confers the plants have the ability to resist to adverse environmental stresses [22]. In our previous study, we proved ROS and ethylene to act the function in regulating the separation of leaf petioles under drought by transcriptomic, physiological and transgenic methods. Moreover, ROS can be increased by the accumulation of proline and polyamine in cassava abscission zones under drought stress [22]. Proline is one of the precursors of polyamine biosynthesis. In our previous research, proline degradation into polyamine was occurred at the abscission zones under drought. Polyamine produced from proline can be depredated into ROS (hydrogen peroxide) by polyamine oxidase gene (PAO) in cassava abscission zones [22]. Under various environmental stresses, GSTs from plant**s** exhibited peroxidase activity and played roles on enhancing tolerance to oxidative stress [1, 2]. Many *GST* genes were identified from various plants [1]; however, no information is available regarding the *GST* family in cassava.

In this study, we identified 83 *GST* family members from the cassava genome, the number of GST genes in cassava genome is a little more than the number in the Populus genome (81 GSTs) [5]. An evolutionary analysis suggested that 83 cassava GSTs could be grouped into 9 subfamilies. Further, the phylogenetic tree and amino acid motifs prediction and analysis were carried out. A globe microarray analysis was used to analyze the *GST*s that presented in cassava abscission zones in both drought and ethylene treatments induced leaf abscission; the comparison analyses of the expression pattern of *MeGST*s between ethylene and drought treatments induced leaf abscission showed the *MeGST*s had similar expression profiles in both treatments. Further research indicated 17 Tau *GST*s are widely up-regulated in the cassava abscission zones by comparison of *GST* expression profiles between ethylene and drought induced leaf abscission. These Tau *GST*s were further identified in two cassava cultivars with different degrees of leaf abscission when suffered from the same drought stress. Together, the data indicate that Tau *GST*s regulate the progression of cassava leaf abscission.

## Methods

### Plant materials and treatments

Cassava cultivars SC124 and Arg7 were planted in plastic pots in greenhouse for six months, the cassava plants grown in greenhouse at 28 °C with a 16 h light photoperiod (130 μmol·m^− 2^·s^− 1^). In one pot, three plants were planted, and three pots were used as a biological replicate [22]. To identify the cassava *GST* genes in abscission zones, 90 days old of cassava abscission zones were collected from cassava genotypes under standard and stress conditions [22]. Chlorophyll fluorescence parameter Fv/Fm [22] was used to evaluate the process of leaf abscission in both drought and ethylene treatments [22]. For ethylene treatment, cassava plants were sprayed with 100 μM ethylene; for drought treatment, the cassava plants grown with no water under green house room. For collection the samples of ethylene and drought treatments, Fv/Fm values were used to collect abscission zone samples at six time points during stresses. To confirm the expression profiles under drought in microarray with real time PCR, samples were cut from cassava plants with three biological replicate pots and repeated 3 times for each time point.

### Identification and evolutionary analyses

GST protein sequences of cassava and *Populus* were obtained from the genomes in the Phytozome (https://phytozome.jgi.doe.gov/pz/portal.html#!info?alias=Org_Mesculenta) and NCBI databases, respectively [22]. To identify the cassava *GST* family genes, known *GST* from *Populus* as a query to search the cassava genome database with local Hidden Markov Model-based searche (https://www.techylib.com/fr/view/powemryologist/stif_hidden_markov_model-based_search_algorithm_for_the); subsequently, the predicted *GST*s from cassava database were evaluated by BLAST searche with homologous GSTs from other plant species. Then, the candidate GST proteins were further analyzed by PFAM (http://pfam.sanger.ac.uk/) database analysis and CDD (http://www.ncbi.nlm.nih.gov/cdd/) analysis. After that, the conserved domains analyses were carried out to confirm the predicted MeGST proteins with multiple sequence alignments. Finally, all GST proteins from cassava and *Populus* alignment analyses were confirmed by Clustal X 2.0 analysis. The bootstrap neighbour-joining analysis was carried out by MEGA 5.0 software.

### Gene motif detection in cassava

The gene motif detection of all 83 cassava MeGSTs was analyzed by the Multiple Em program from the Motif Elicitation (MEME; version 4.9.0) tool, the parameters of the conserved motifs in all cassava *GST* proteins were identified as previously described [22, 23]. InterProScan (http://www.ebi.ac.uk/Tools/pfa/iprscan/) were performed to annotate the motifs.

### Transcriptome analysis

The microarray analyses for either ethylene or drought treatment were carried out as previously described [22]. The abscission zones samples of drought and ethylene treatments were collected for total RNA extraction with a plant RNeasy extraction kit (TIANGEN, Beijing, China) for transcriptome analysis [22]. The samples were repeated for 3 times, and the replicate samples were separately sequenced. The transcriptome analyses were performed as previously described [22]. For microarray data analysis, the up or down regulated *GST* genes exceeding a threshold fold change > 2.0 or < 0.5 (log base 2), a Wilcoxon Rank-Sum test significance level at 0.05 (*P* < 0.05) were considered significant.For Quantitative real-time PCR analysis, the relative expression was used to elevate the transcript levels of the candidate genes. The up or down regulated *GST*s were grouped by Hierarchical clustering with the MeV 4.0 software. GO annotation of Tau *MeGST*s genes was carried out by BiNGO according to Maere et al., (2005) [24].

### Quantitative real-time PCR analysis

RNA from three independent biological cassava abscission zone samples under drought or ethylene treatments were used for real-time qRT-PCR on a STEP-ONE system with SYBR Green I (Carlsbad, CA) detection. The real-time qRT-PCR performed as previously described [22]. The primer sequences for real-time PCR are listed in Additional file 1 Data 1.

## Results

### Cassava glutathione S-transferase gene identification and phylogenetic reconstruction

**Fig. 1:**
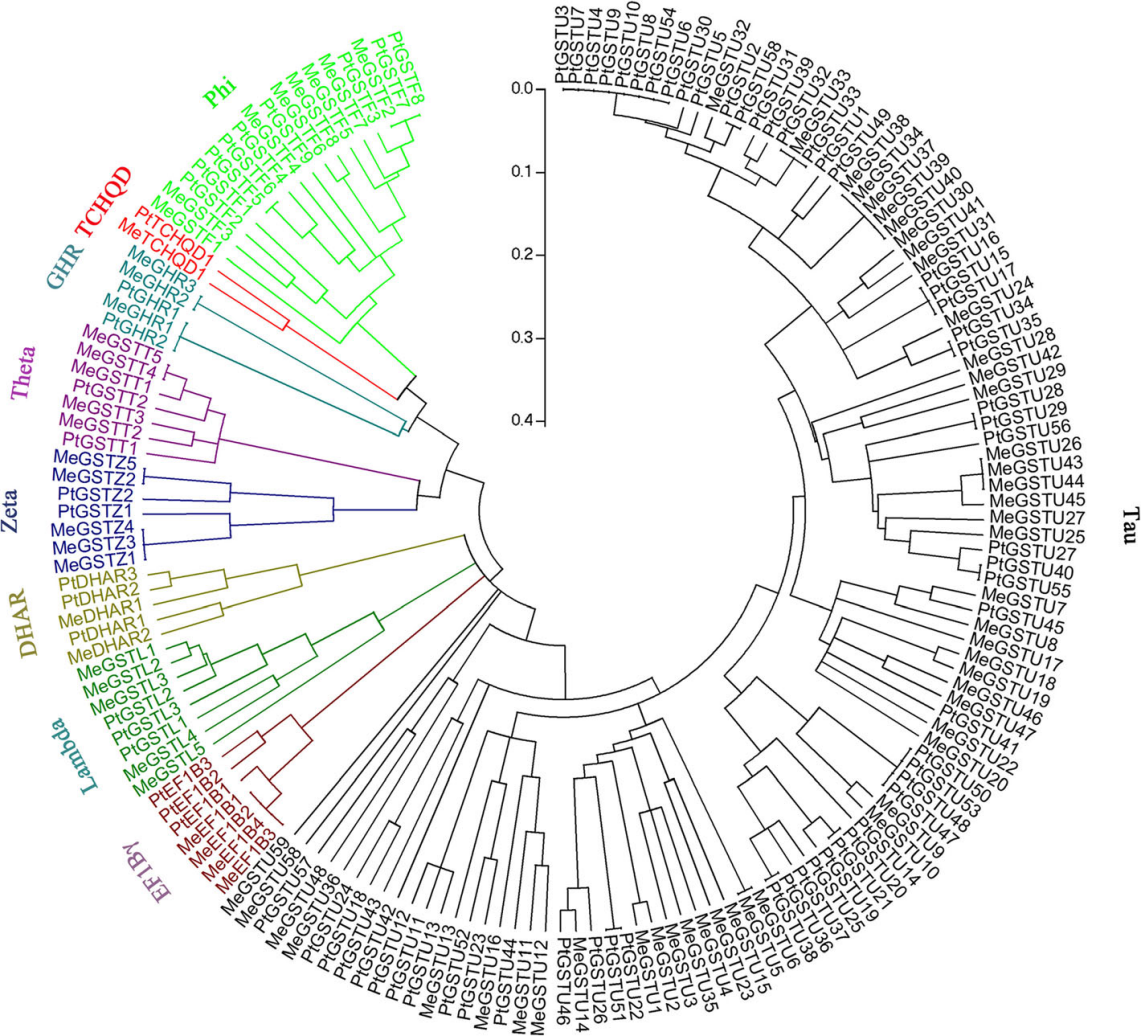
Phylogenetic tree construction of all GSTs from cassava and *Populus*. 83 cassava GSTs and 81 *Populus* GSTs were analyzed using ClustalW, and the phylogenetic tree was constructed by MEGA 5.0 software using the neighbour-joining method (based on the p-distance model with 1000 bootstrap replicates). Each group is represented by a specific colour

A total of 83 cassava *GST*s (Additional file 2: Data 2) were identified from the cassava genome (annotation v. 6.1) with a BLAST search of the cassava genome database with homologous GST coding sequences from plant species as queries. The gene set represents approximately 0.2513% (83/33,033) of the annotated genes in cassava genome (33,033 genes), which is a little bigger than the proportion of *Arabidopsis* genome (0.2006%) [25–28]. To study the phylogenetic relationships of the GST genes between cassava and *Populus*, all identified cassava GSTs and those from *Populus* were analyzed with the multiple sequence alignment analysis. A phylogenetic tree including GSTs form cassava (83) and *Populus* (81) was generated (Fig. 1). The resulting phylogenetic tree contained 9 classes, termed Phi, Tau, Theta, Zeta, Lambda, DHAR, TCHQD, EF1Bγ and GHR classes. All of the classes contained *GST* from cassava and *Populus* genomes, some of these members from both *Populus* and cassava can be clustered together, suggesting that these members may act the same function in plant development. The Tau GST class showed have the maximum number of members, including 59 members came from cassava and 58 from *Populus*, respectively. The minimum number of members was TCHQD class, only 1 member from cassava and 1 member from *Populus* genome.

### Phylogenetic and conserved gene structure and protein motif analysis of *GST* gene families in cassava

**Fig. 2:**
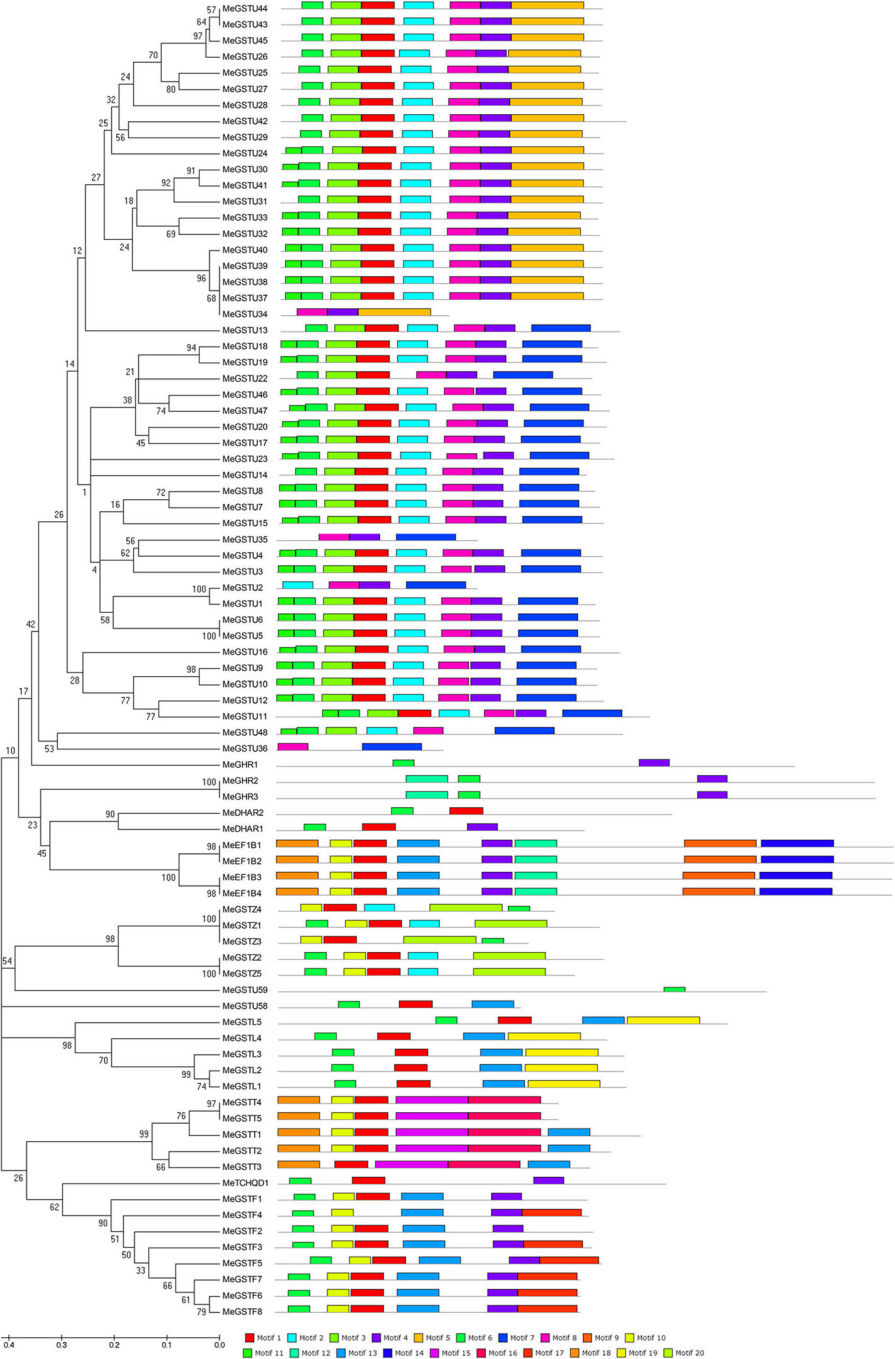
Conserved motifs of cassava GST proteins by the evolutionary relationship analysis. MEME was used to identify the conserved motifs in the MeGST proteins. Each motif is indicated by a coloured box numbered at the bottom, and grey lines represent the non-conserved sequences. All identified motifs were annotated with the help of InterProScan (http://www.ebi.ac.uk/Tools/pfa/iprscan/)

To confirm the conserved protein motifs in all the GST proteins, the Multiple Em program for the Motif Elicitation (MEME; version 4.9.0) tool analysis was carried out to evaluate all the 83 MeGST proteins. 20 conserved protein motifs in the MeGSTs were detected by MEME software analysis and the motif annotation was analyzed with InterPro analysis. The motif lengths with 11 and 50 amino acids long were confirmed by MEME analysis (Fig. 2; Additional file 3: Figure S1). In detail, most of the Phi *GST* genes have motifs 4, 6, 10 and 13; most of the Tau *GST* genes have motifs 1, 2, 3, 4, and 8; most of the Theta *GST* genes have motifs 1, 15, 16 and 18; most of the Zeta *GST* genes have motifs 1, 10 and 20; most of the Lambda *GST* genes have motifs 1, 6 and 13; the DHAR *GST* genes have motifs 1, and 6; the TCHQD *GST* genes have motifs 1, 4, and 6; the GHR *GST* genes have motifs 4 and 6; the EF1B*γ GST* genes have motifs 1, 4, 9, 10, 12, 13, 14, and 18.

### The identification of *GST* genes that presented in cassava abscission zones in both ethylene and drought treatments

To identify the *MeGST* gene expression patterns in both ethylene and drought induced cassava leaf abscission, six time points were determined in both treatments by cassava leaf Fv/Fm values detection described as our previous research [22]. To confirm the gene expression patterns in cassava abscission zones in both ethylene and drought induced leaf abscission, a whole genome microarray (NimbleGen) of cassava was constructed and analyzed as our previous research [22].

**Fig. 3:**
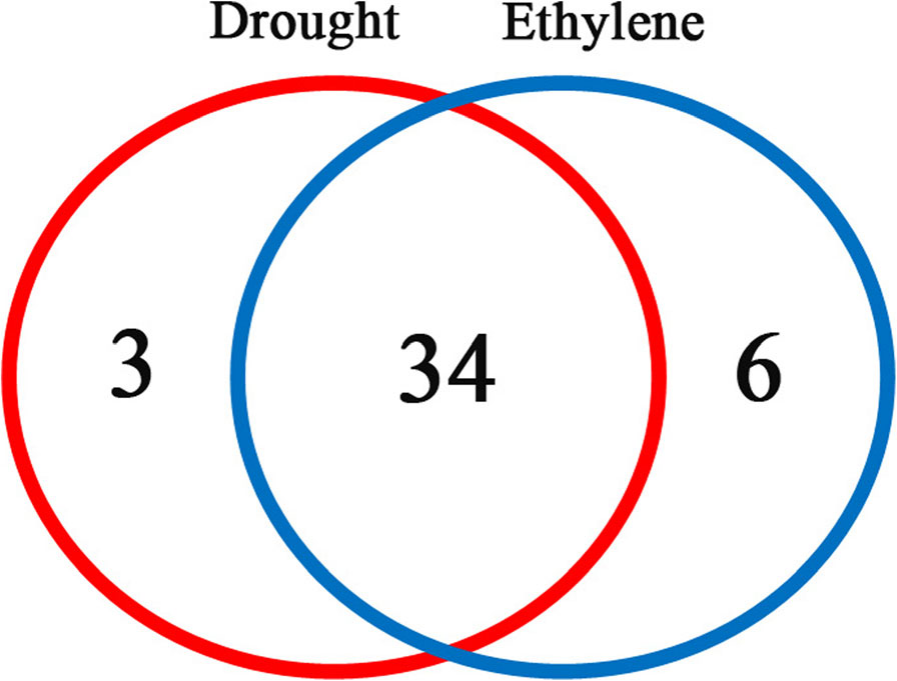
*MeGST*s shared between and unique to ethylene and drought induced leaf abscission

In the data of microarray, 40 *GST*s were detected with different expression patterns in ethylene induced leaf abscission, while 37 *GST* genes were differentially expressed in drought induced leaf abscission (Additional file 4: Data 3). 34 *GST* genes were detected with co-expression in both ethylene and drought induced leaf abscission (Fig. 3). 6 *GST* genes were expressed only in ethylene induced leaf abscission, i.e. *MeGSTL1, MeGSTU10, MeGSTU16, MeGSTU24*; *MeMAPEG1, MeMAPEG2*; 4 *GST* genes were expressed only in drought induced leaf abscission, i.e. *MeGSTF7, MeGSTF8, MeGSTL1* (Fig. 3, Additional file 4: Data 3).

**Fig. 4:**
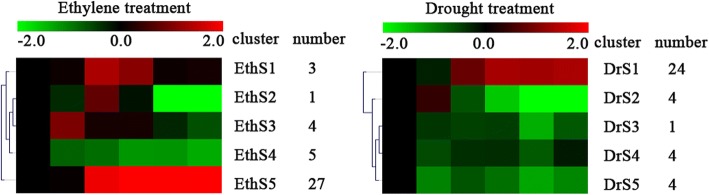
SOTA clustering showing the expression profiles of ethylene and drought induced leaf abscission. Five clusters of *GSTs* gene expression patterns at the six time points during leaf abscission were identified by SOTA clustering analysis (40 and 37 *GST* genes for ethylene and drought induced leaf abscission, respectively). A red-green colour scale represented the *GST* genes expression signals; where red and green represent up-regulated and down-regulated expression, respectively

In all 40 *MeGST* genes differentially expressed in ethylene induced leaf abscission, all 40 *MeGST* genes were analyzed by SOTA analysis, and five clusters (EthS1-EthS5) were grouped by SOTA analysis, moreover, five clusters were classified into four groups of expression patterns (Fig. 4 and Additional file 5: Data 4). The first group (clusters EthS5) represents the *GST* genes that were up-regulated in the whole experimental period compared with the T1 time point control in ethylene induced leaf abscission, 27 genes grouped into this expression patterns. The second group included the genes shown in cluster EthS3, exhibiting the genes up-regulated in T2 time point, 4 genes showed the expression pattern in this group. The third group (clusters EthS1 and EthS2) showed the genes up-regulated in T3 and T4 time points, 4 genes grouped into this expression pattern. The fourth group (cluster EthS4) exhibited the genes that down-regulated throughout the experimental period, 5 genes were grouped into this expression pattern.

In 37 *GST* genes expressed in drought induced leaf abscission, five clusters (DrS1-DrS5) were grouped by SOTA analysis, and five clusters could be classified into three classes of expression profilings (Fig. 4 and Additional file 5: Data 4). The first class (clusters DrS1) exhibited the genes that up-regulated in the whole experimental period while compared with the T1 time point, 24 genes were grouped into this expression pattern. The second group (cluster DrS2) showed the genes that up-regulated in the T2 time point, 4 genes were classed into this expression pattern. The third group (cluster DrS3, DrS4 and DrS5) exhibited the genes that down-regulated in the whole experimental period, 9 genes were classed into this expression pattern.

### Comparison of *GST* expression profiles between ethylene and drought induced leaf abscission indicated that tau *GST*s are widely up-regulated in the cassava abscission zones

The comparison of *MeGST* expression profiles in both ethylene and drought induced leaf abscission were carried out by SOTA clustering to confirm the *GST* genes that participate in both treatments. So the similar expression profiles of *GST*s at each time point in both ethylene and drought treatments were analyzed.

We first examined the *GST* genes that were up-regulated in the whole experimental period compared with the T1 time point in response to both treatments (Fig. 4 and Additional file 1: Data 1). The expression profiles of genes in EthS5 in ethylene treatment and DrS1 in drought treatment were first compared. 27 genes in ethylene treatment were grouped into this group, GO annotation indicated that 10 genes participated in the pathway of toxin catabolism (GO: 0009407), 4 genes participated in the pathway of response to cadmium ion (GO: 0046686), 2 genes participated in the pathway of response to oxidative stress (GO: 0006979). 24 genes in drought treatment were classed into this group, GO annotation indicated that 10 genes also participated in the pathway of toxin catabolism (GO: 0009407), 4 genes participated in the pathway of response to cadmium ion (GO: 0046686), 2 genes participated in the pathway of response to oxidative stress (GO: 0006979). 19 *GST* genes were detected with similar expression patterns in both treatments, i.e. *MeGSTF3, MeGSTF6, MeGSTL2, MeGSTU11, MeGSTU13, MeGSTU14, MeGSTU18, MeGSTU19, MeGSTU20, MeGSTU27, MeGSTU3, MeGSTU30, MeGSTU34, MeGSTU35, MeGSTU5, MeGSTU6, MeGSTU7, MeGSTU9* and *MeGSTZ2*. GO annotation indicated that 9 genes participated in the pathway of toxin catabolism (GO: 0009407), 4 genes participated in the pathway of response to cadmium ion (GO: 0046686), 2 genes participated in the pathway of response to oxidative stress (GO: 0006979).

**Fig. 5:**
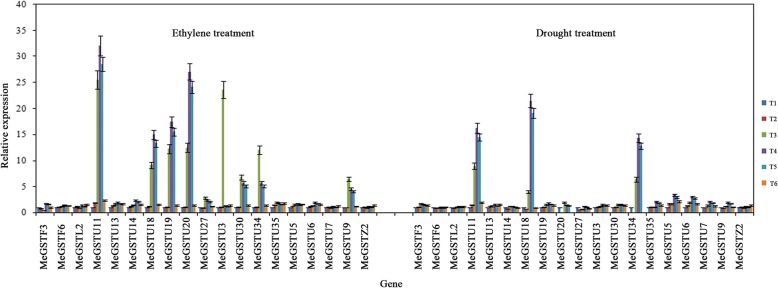
Expression patterns of 19 *MeGST* genes in abscission zones with the similar expression patterns of microarray data confirmed by quantitative real-time PCR under ethylene and drought induced leaf abscission. The relative expression levels of *MeGSTs* were compared with T1 as a control. Data are the means ± SD calculated from three biological replicates

The 19 GST genes have similar expression pattern in both treatment, however, the levels of up-regulation were different in both treatments, more genes with higher expression levels (> 5 times expression levels compared with control) in ethylene treatment were detected while compared with the genes in drought treatment. In ethylene treatment, *MeGSTU18, MeGSTU20, MeGSTU11, MeGSTU19, MeGSTU3, MeGSTU30, MeGSTU34* and *MeGSTU9* were detected with high expression levels under ethylene induced leaf abscission; while in drought treatment, only *MeGSTU18, MeGSTU11* and *MeGSTU34* were examined with high expression levels under drought induced leaf abscission. Quantitative real-time PCR also confirmed these *MeGST*s expressed with high levels under ethylene and drought treatments based on microarray data (Fig. 5).

At the early stage of leaf abscission (T2), the EthS3 cluster in ethylene treatment and DeS2 cluster in drought treatment showed similar expression profiles in both treatments at T2 time point (Fig. 4). 4 genes, *MeGSTZ1*, *MeGSTU28*, *MeGSTT1* and *MeGSTU25*, that showed with high expression levels at this time point in ethylene treatment, GO annotation suggested that all the *GST* genes participated in the pathway of toxin catabolism (GO: 0009407); 4 genes, *MeGSTZ1*, *MeGSTU28*, *MeGSTF4* and *MeGSTF5*, that exhibited with high expression levels at this point in drought treatment. GO annotation indicated that all the *GST* genes participated in the pathway of toxin catabolism (GO: 0009407). 2 *GST* genes were discovered with similar expression profiles in both treatments, i.e. *MeGSTZ1* and *MeGSTU28*.

The EthS4 cluster in ethylene treatment and DrS3, DrS4 and DrS5 clusters in drought treatment indicated that the genes down-regulated throughout the experimental period. 5 genes expressed in ethylene treatment were classed into this group, i.e. *MeGSTL4, MeGSTT3, MeGSTU32, MeGSTU33* and *MeGSTF5,* GO annotation indicated that 4 *GST* genes participated in the pathway of toxin catabolism (GO: 0009407); 9 genes expressed in drought treatment were grouped into this group, i.e. *MeGSTU12*, *MeGSTT1, MeGSTL1, MeGSTT2, MeGSTT3, MeGSTL4, MeGSTU33, MeGSTU25* and *MeGSTL3*, GO annotation indicated that 4 *GST* genes participated in the pathway of toxin catabolism (GO: 0009407). 3 *GST* genes were discovered with similar expression profiles in both treatments, i.e. *MeGSTL4, MeGSTT3,* and *MeGSTU33*.

As described above, 24 *GST* genes were detected with the similar expression across the time points of leaf abscission in both treatments, and 17 Tau *MeGST* genes have the similar expression in both treatments, i.e. *MeGSTU11, MeGSTU13, MeGSTU14, MeGSTU18, MeGSTU19, MeGSTU20, MeGSTU27, MeGSTU3, MeGSTU30, MeGSTU34, MeGSTU35, MeGSTU5, MeGSTU6, MeGSTU7, MeGSTU9*, *MeGSTU28* and *MeGSTU33*, suggesting that most of the Tau *GST* genes played similar role in regulation leaf abscission in environment-induced leaf abscission.

### Validation of gene expression patterns of seventeen tau *GST*s in two cultivated varieties, *Arg7* and *SC124* with different levels of leaf abscission under the same drought condition

**Fig. 6:**
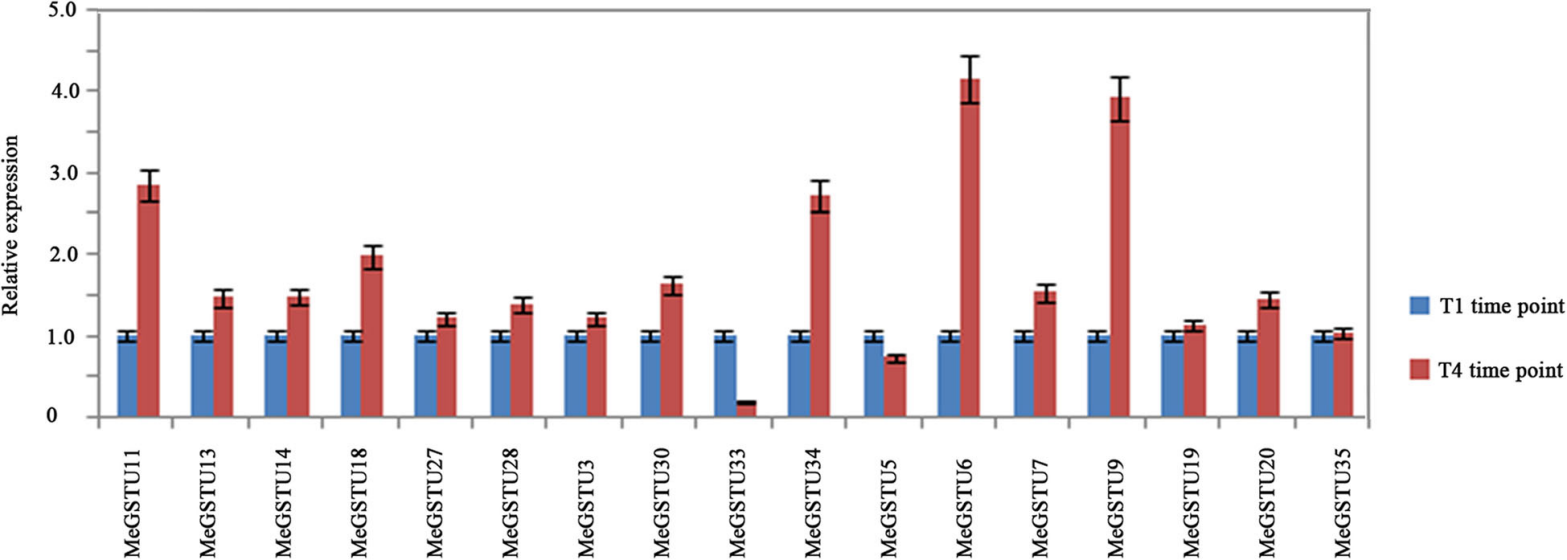
Identification of 17 Tau *MeGST* genes in two cassava cultivars (‘SC124’ and ‘Arg7’) under drought. The expression ratios for each Tau *MeGST* gene at each time point are presented first using T1 as a control for both ‘Arg7’ and ‘SC124’, and then to cut ‘Arg7’ expression ratios with those of ‘SC124’ at each time point

To further study the function of Tau *GST*s in cassava leaf abscission, the expression patterns of these 17 Tau *GST*s were detected in two cassava cultivars, ‘Arg7’ and ‘SC124’. The two cassava cultivars exhibit the different levels of leaf abscission under the same drought condition. Compared with cultivar SC124, cultivar Arg7 is more easily shed the leaves than SC124 cultivar when met the same drought condition [23]. In our previous research, the middle stage at drought stress is the important stage for determining cassava leaf abscission [22], so we chosen T4 time point as the time point to analyze the GST genes expression in two cultivars [22]. The expression patterns of the 17 *GSTU*s were analyzed in two cultivars at T4 time point with T1 as control under drought stress. The results showed that all the 17 *GSTU* genes were detected with expression at T4 time point in drought treatment, 15 genes detected with higher level expression in Arg7 than that in SC124 at T4 time point, while 2 genes, *MeGST33* and *MeGST5*, showed the lower level expression in Arg7 than that in SC124 at T4 time point (Fig. 6). *MeGSTU34, MeGSTU6, MeGSTU11, MeGSTU18* and *MeGSTU9* detected with high expression level under drought compared with the other genes. The maximum expression ratios were approximately 3-, 4-, 3-, 2- and 4-fold up-regulated in Arg7 when compared in SC124 (Fig. 6).

## Discussion

### The *GST*s have similar expression patterns in cassava abscission zones under various stresses induced leaf abscission

The GSTs family played important regulation role in enhancing tolerance to oxidative stress, osmotic dehydration, and plant hormones [1, 2]. In this study, we identified 83 *GST* family members from the cassava genome. Here, 83 cassava GST genes were grouped into 9 classes (Fig. 1). Previously, Dixon et al. reported 55 *AtGST* genes in *Arabidopsis* genome. The significant expression patterns of *GSTs* were detected in ethylene and drought treatments induced leaf abscission in cassava abscission zones, similar genes (34 *GSTs* in all 83 *GST* genes) were induced expression in cassava abscission zones in both ethylene and drought treatments induced leaf abscission, suggesting the important function for these *GST* genes in cassava leaf abscission regulation, the similar expression profiles of *GSTs* in cassava abscission zones under various stresses induced leaf abscission, indicating that most *GST* genes may play important regulation roles in abscission zones development under stresses.

### Tau MeGSTs might contribute to the robust resistance to alleviate ROS accumulation in cassava abscission zones that produced by various stresses induced leaf abscission

17 Tau *MeGSTs* showed the similar expression profiles in both ethylene and drought induced leaf abscission; this suggests that the 17 *MeGSTU* genes have the same function in regulating cassava abscission zones growth and development when suffering from ethylene and drought stresses. The primary function of GSTUs is detoxification. In our study, many *MeGSTUs* were detected to express in both treatments induced leaf abscission, and GO annotation suggested that most of the *GSTUs* participated in the pathway of toxin catabolism (GO: 0009407), suggesting that these genes may detoxify the ROS that produced during both ethylene and drought treatments induced leaf abscission in cassava. We previously demonstrated that ROS accumulated in cassava abscission zones during drought stress [22]. In this study, we discovered that most of the *MeGSTUs* up-regulated in abscission zones under drought and ethylene treatments in cassava, GO annotation indicated most of the *MeGSTUs* up-regulated in abscission zones in both treatments can participate in the pathway of ROS, suggesting the *MeGSTUs* up-regulated in abscission zones may involved in regulation the adverse stresses resistance in cassava abscission zones by regulating ROS accumulation under stresses. Substantial evidence has confirmed that *GSTUs* play regulation roles in alleviating ROS accumulation that produced under drought stress in plants [18]. GSTUs are proved to involve in the response to various forms of oxidative stress, such as salt, heavy metals, drought, phytohormone and others [20]. The increase in GSTU levels can be induced by excessive ROS [20]. In cassava, abundant ROS accumulate when the plants suffer from environmental stresses [22]; the ROS in the plants promote the expression of the *MeGSTU*s. It suggested that the high expression levels of *MeGSTs* in abscission zones induced by drought or ethylene treatments in cassava may confer the genes to enhance the tolerance to ROS that induced by stresses in cassava plants [29, 30].

Many *GSTU* were found to participate in the stress response in *Arabidopsis*, and the function of the *GSTU* that reported can regulate the level of proline and antioxidant enzymes proved by stresses in plants [2], additionally, the increase levels of proline and antioxidant enzyme activity in the overexpression *GSTU* transgenic plants that contribute the plants to promote the drought tolerance under drought. As described in our previous research, proline and antioxidant enzymes were proved to involve in the ROS accumulation and regulate leaf abscission in cassava under drought [22]. These findings indicated that *GSTU* regulate the levels of proline and antioxidant can enhance resistance to stress tolerance in cassava. We also discovered high levels of proline in cassava induced by drought [22]. Thus, *MeGSTU* may regulate stress response in cassava by regulating the levels of proline and antioxidant enzymes. In short, these findings suggested that *MeGSTs* may contribute to confer the cassava plants with robust resistance to ROS that produced by environmental stresses.

## Conclusion

In conclusion, in this study, we first identify and analyze the GST gene family in cassava abscission zone. Here, 83 cassava GST genes were grouped into 9 classes, the expression of *MeGST*s was identified and characterized in different environmental stresses induced leaf abscission, and the stress-related *MeGST*s were also discussed in different cultivated varieties. The hypothesis was got that Tau *MeGST*s may be found to be responsible for alleviating ROS accumulation under various stresses induced leaf abscission in cassava abscission zones.

## Additional files

Additional file 1: **Data 1.** Primers used in qRT-PCR analysis. (XLS 39 kb)

Additional file 2: **Data 2.** The accession numbers of *GST*s in cassava. (XLSX 20 kb)

Additional file 3: **Figure S1.** Sequence logos for conserved motifs identified in MeGSTs by MEME analysis. (JPG 1160 kb)

Additional file 4: **Data 3.** Cassava *GST* genes expressed in abscission zones in ethylene treatment or drought treatment induced leaf abscission. (XLSX 18 kb)

Additional file 5: **Data 4.** Five clusters of the cassava *GST* genes expressed in abscission zones in ethylene treatment or drought treatment induced leaf abscission by Hierarchical clustering analysis. (XLS 36 kb)

## Abbreviations

Fv/Fm: Chlorophyll fluorescence parameter
GO: Gene ontology
GSH: Tripeptide glutathione
GST: Glutathione S-transferases
ROS: Reactive oxygen species
SOTA: Self-organizing tree algorithm

## References

[1] Dong Y, Li C, Zhang Y, He Q, Daud MK, Chen J, Zhu S (2016). Glutathione S-transferase gene family in *Gossypium raimondii* and *G. arboreum*:comparative genomic study and their expression under salt stress. Front Plant Sci.

[2] Xu J, Tian YS, Xing XJ, Peng RH, Zhu B, Gao JJ, Yao QH (2015). Over-expression of AtGSTU9 provides tolerance to salt, drought and methyl viologen stresses in *Arabidopsis*. Physiol Plant.

[3] Lallement PA, Brouwer B, Keech O, Hecker A, Rouhier N (2014). The still mysterious roles of cysteine-containing glutathione transferases in plants. Front Pharmacol.

[4] Labrou NE, Papageorgiou AC, Pavli O, Flemetakis E (2015). Plant gstome:structure and functional role in xenome network and plant stress response. Curr Opin Biotechnol.

[5] Lan T, Yang ZL, Yang X, Liu YJ, Wang XR, Zeng QY (2009). Extensive functional diversification of the populus glutathione s-transferase supergene family. Plant Cell.

[6] Sappl PG, Carroll AJ, Clifton R, Lister R, Whelan J, Harvey MA, Singh KB (2009). The Arabidopsis glutathione transferase gene family displays complex stress regulation and co-silencing multiple genes results in altered metabolic sensitivity to oxidative stress. Plant J.

[7] Soranzo N, Gorla MS, Mizzi L, Toma GD, Frova C (2004). Organisation and structural evolution of the rice glutathione S-transferase gene family. Mol Genet Genomics.

[8] Jain M, Ghanashyam C, Bhattacharjee A (2010). Comprehensive expression analysis suggests overlapping and specific roles of rice glutathione S-transferase genes during development and stress responses. BMC Genomics.

[9] Rezaei MK, Shobbar ZS, Shahbazi M, Abedini R, Zare S (2013). Glutathione S-transferase (GST) family in barley:identification of members, enzyme activity, and gene expression pattern. J Plant Physiol.

[10] Licciardello C, D’Agostino N, Traini A, Recupero GR, Frusciante L, Chiusano ML (2014). Characterization of the glutathione S-transferase gene family through ESTs and expression analyses within common and pigmented cultivars of Citrus sinensis (L.) Osbeck. BMC Plant Biol.

[11] Horváth E, Bela K, Papdi C, Gallé Á, Szabados L, Tari I, Csiszár J (2015). The role of Arabidopsis glutathione transferase F9 gene under oxidative stress in seedlings. Acta Biol Hung.

[12] Kouno T, Ezaki B (2013). Multiple regulation of Arabidopsis AtGST11 gene expression by four transcription factors under abiotic stresses. Physiol Plant.

[13] Bianchi MW, Roux C, Vartanian N (2002). Drought regulation of GST8, encoding the*Arabidopsis* homologue of ParC/Nt107 glutathione transferase/peroxidase. Physiol Plant.

[14] Dixon DP, Davis BG, Edwards R (2002). Functional divergence in the glutathione transferase superfamily in plants identification of two classes with putative functions in redox homeostasis in *Arabidopsis thaliana*. J Biol Chem.

[15] Jiang HW, Liu MJ, Chen IC, Huang CH, Chao LY, Hsieh HLA (2010). Glutathione*S*-transferase regulated by light and hormones participates in the modulation of Arabidopsis seedling development. Plant Physiol.

[16] Mang HG, Kang EO, Shim JH, Kim SY, Park KY, Kim YS, Bahk YY, Kim WT (2004). A proteomic analysis identifies glutathione *S*-transferase isoforms whose abundance is differentially regulated by ethylene during the formation of early root epidermis in *Arabidopsis* seedlings. Biochim Biophys Acta.

[17] Øverby A, Stokland RA (2013). Åsberg SE Sporsheim B, bones AM. Allyl isothiocyanate depletes glutathione and upregulates expression of glutathione s-transferases in arabidopsis thaliana. Front Plant Sci.

[18] Sharma R, Sahoo A, Devendran R, Jain M (2014). Over-expression of a Rice tau class glutathione S-transferase gene improves tolerance to salinity and oxidative stresses in Arabidopsis. PLoS One.

[19] Chan C, Lam HM (2014). A putative lambda class glutathione S-transferase enhances plant survival under salinity stress. Plant Cell Physiol.

[20] Liu D, Liu Y, Rao J, Wang G, Li H, Ge F, Chen C (2013). Overexpression of the glutathione S-transferase gene from *Pyrus pyrifolia* fruit improves tolerance to abiotic stress in transgenic tobacco plants. Mol Biol.

[21] Jha B, Sharma A, Mishra A (2011). Expression of *SbGSTU* (tau class glutathione S-transferase) gene isolated from *Salicornia brachiata* in tobacco for salt tolerance. Mol Biol Rep.

[22] Liao W, Wang G, Li Y, Wang B, Zhang P, Peng M (2016). Reactive oxygen species regulate leaf pulvinus abscission zone cell separation in response to water-deficit stress in cassava. Sci Rep.

[23] Hu W, Yang H, Yan Y, Wei Y, Tie W, Ding Z, Zuo J, Peng M, Li K (2016). Genome-wide characterization and analysis of bZIP transcription factor gene family related to abiotic stress in cassava. Sci Rep.

[24] Maere S, Heymans K, Kuiper M (2005). BiNGO:Cytoscape plugin to assess overrepresentation of gene ontology categories inbiological networks. Bioinformatics.

[25] Marrs KA. The function and regulation of glutathione-S-transferase in plants. Annual Review of Plant Biology. 1996;47:127–58. 10.1146/annurev.arplant.47.1.127 15012285

[26] Sappl PG, Onate-Sanchez L, Singh KB, Millar AH (2004). Proteomic analysis of glutathione S-transferases of Arabidopsis thaliana reveals differential salicylic acid-induced expression of the plant-specific phi and tau classes. Plant Mol Biol.

[27] Wagner U, Edwards R, Dixon DP, Mauch F (2002). Probing the diversity of the Arabidopsis glutathione S-transferase gene family. Plant Mol Biol.

[28] Dixon DP, Edwards R (2010). Glutathione Transferases. The Arabidopsis Book/American Society of Plant Biologists.

[29] You J, Chan Z (2015). ROS regulation during abiotic stress responses in crop plants. Front Plant Sci.

[30] Miller G, Suzuki N, Ciftci-Yilmaz S, Mittler R (2010). Reactive oxygen species homeostasis and signalling during drought and salinity stresses. Plant Cell Environ.

