# Supplementary figures and images for: Tau GSTs involved in regulation of leaf abscission by comparison the gene profiling of MeGSTs in various abscission-promoting treatments in cassava abscission zones
Source: BMC Genet. 2018 Jul 13;19:45. doi: 10.1186/s12863-018-0627-6 (PMC6043963; doi:10.1186/s12863-018-0627-6)

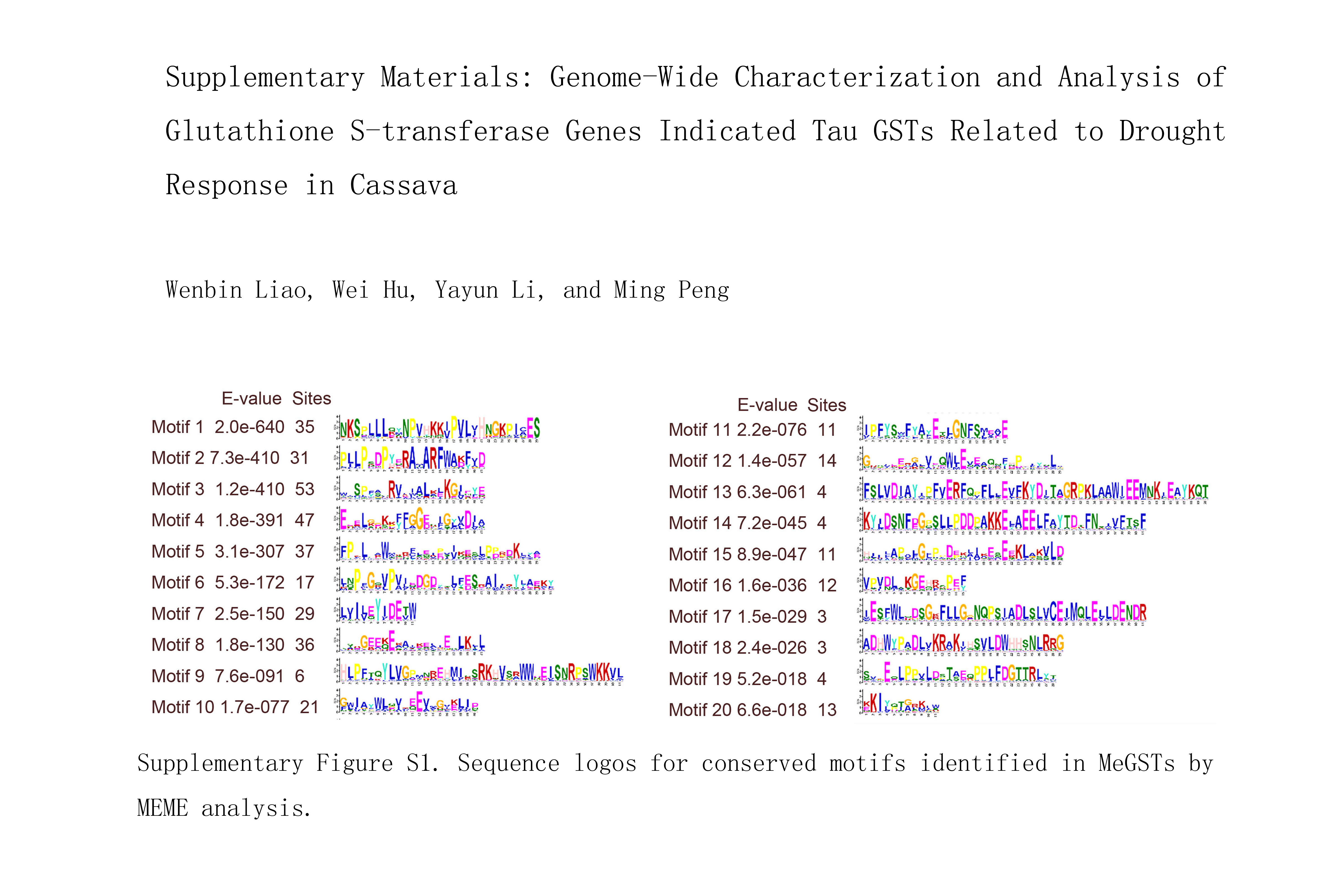

Supplement: Supplementary file 3 — Figure S1. Sequence logos for conserved motifs identified in MeGSTs by MEME analysis. (JPG 1160 kb) [file 12863_2018_627_MOESM3_ESM.jpg]
